# The Uterine Environment and Childhood Obesity Risk: Mechanisms and Predictions

**DOI:** 10.1007/s13668-023-00482-z

**Published:** 2023-06-20

**Authors:** Andreea Cristian, Jane L. Tarry-Adkins, Catherine E. Aiken

**Affiliations:** 1 Department of Obstetrics and Gynaecology, University of CambridgeThe Rosie HospitalandNIHR Cambridge Biomedical Research Centre, Box 223, Cambridge, CB2 0SW UK; 2 Wellcome-MRC Institute of Metabolic Science and Medical Research Council Metabolic Diseases Unit, University of Cambridge, Addenbrooke’s Hospital, Cambridge, CB2 0QQ UK

**Keywords:** Intrauterine environment, Developmental programming, Maternal, Childhood, Obesity, Interventions

## Abstract

**Purpose of Review:**

Childhood obesity is a growing health problem in many populations, hence the urgent need to unravel the underlying mechanisms. Some evidence suggests that exposure to suboptimal intrauterine environments can program foetal metabolic health, with adverse consequences in later life, including susceptibility to childhood obesity.

**Findings:**

Factors such as high and low foetal birth weight, excessive gestational-weight-gain, maternal stress and smoking are all associated with increased risk of childhood obesity in observational studies. Animal models, where both genetic background and the postnatal environment can be carefully controlled, suggest that several different mechanisms, including epigenetic changes, dysregulation of adipose tissue development and programming of appetite, may be key drivers of developmental programming of childhood obesity. However, the influence of genetics and the post-natal environment are much more difficult to disentangle as independent effects in human studies, which are also complicated by low follow-up rates.

**Summary:**

Suboptimal intrauterine environments interact with maternal and foetal genetics and with the postnatal environment to contribute to the risk of childhood obesity. Maternal metabolic challenges, for example obesity and insulin resistance, contribute to the risk of foetal overgrowth and subsequent adiposity in childhood. To protect the long-term health of populations, research focusing on effective means of identifying and intervening in the transgenerational cycle of childhood obesity is required.

## Introduction

Current predictions suggest that by 2030, more than 250 million children worldwide will be living with obesity [1]. In many global areas, rates of childhood obesity continue to increase [1]. Longitudinal data suggests that children affected by obesity have > 80% risk of remaining affected into adulthood [2] and are thus at risk of a multitude of adverse health outcomes. It is therefore an urgent research priority to understand the mechanisms underlying childhood obesity, identify children at risk early in life and to develop effective interventions to prevent obesity. Tracing the origins of childhood obesity back to intrauterine development allows the identification of opportunities for intervention early in the pathway.

A major difficulty in determining the mechanisms by which the intrauterine environment can program childhood obesity is disentangling the impact of a suboptimal intrauterine environment from other key influences, including shared maternal and foetal genetics, and the impact of the post-natal environment. Genetic influences on childhood obesity include those where the causal association is straightforward, for example monogenic mutations in the melanocortin 4 receptor [3]. However, these cases comprise only ~ 2–3% of childhood obesity [4], with polygenic influences being more relevant in the majority of cases. Childhood BMI can be influenced both by the direct impacts of foetal genetic risk and the indirect impact of maternal genetic risk for obesity, an association that may also be at least in part mediated via the intrauterine environment.

The influence of genetics is complicated by interactions with environmental factors, and these can be challenging to unravel [5]. For example, the impact of maternal genetic risk score for body mass index (BMI) on offspring obesity is mediated by the presence or absence of gestational diabetes, illustrating the complexities of gene × environment interactions in determining phenotype [6].

The influence of the postnatal environment on the risk of childhood obesity can also be challenging to delineate. Data from animal models suggests that there are significant interactive effects of exposure to both antenatal and postnatal obesogenic environments [7], but in human cohorts, the relative influence of the antenatal and postnatal periods on childhood obesity is much more difficult to delineate. In the majority of cases, the mother’s antenatal environment and the family postnatal environment have similar risk profiles for development of obesity, for example food types consumed. The best evidence for an independent impact of the antenatal environment comes from sibling pairs born to the same mother before and after a significant change in the intrauterine environment, e.g., in a Swedish study, children born from pregnancies during which their mothers underwent an extensive program of lifestyle interventions intended to limit gestational weight gain (GWG) had lower BMI aged 5 than their siblings born from subsequent non-intervention pregnancies [8]. However, the impact of the postnatal environment is clearly seen in recent studies of sibling pairs born to the same mothers before and after bariatric surgery. These studies show that while pregnancy outcomes are improved following maternal weight loss, there is no sustained lowered risk of childhood obesity in the post-surgery siblings by pre-school age [9], potentially due to family eating behaviours, particularly consumption of sweeteners [10].

Thus, the intrinsic overlapping nature of shared genetics, antenatal and postnatal environments makes confounding influences very difficult to rule out in human studies linking the intrauterine environment with later childhood obesity. Causal links between suboptimal intrauterine environments and adiposity in childhood are clearly suggested by animal studies, but their interpretation is not straightforward in the much more complex context of real-world variation in human behaviours and genetics.

## Evidence from Human Cohorts for the Influence of the Intrauterine Environment

Evidence suggests that patterns of growth in utero correlate with identifiable patterns of growth in postnatal life, for example the frequently observed phenomenon of perinatal catch-up growth. Low estimated foetal weight followed by catch-up growth is associated with increased fat mass at 6 months [11] and at 2 years [12]. The final outcome of foetal growth is birth weight, and it is striking that both high and low birth weights are associated with childhood obesity [13, 14], suggesting that multiple different foetal growth patterns may increase the risk of later adiposity.

Associations between maternal obesity, either before conception [15, 16] or during pregnancy [17, 18], and the risk of childhood obesity are commonly observed in cohort studies. The relationship between maternal BMI and adiposity at birth may also be independent of other important factors such as glycaemic control [19]. Static measures of maternal BMI allow little insight into the contribution of the intrauterine environment to this association; however, dynamic measurements of maternal weight gain specifically during pregnancy may be more informative. Correlations between excessive GWG and childhood obesity have been observed in several studies [20–22]. This observation is caveated by the fact that normal parameters of GWG are poorly defined [23] and that the increased risk of childhood obesity specifically attributable to GWG in mothers with overweight/obesity may be relatively small [24]. The reason for this caution is demonstrated in a large (*n* > 14,000) Californian study, where high GWG was associated with a ~ 30% increased risk of obesity at 3 years [25]. However, when sibling-controlled analysis was applied to the cohort (hence controlling for at least some variation attributable to shared genetics and postnatal environment), the association between GWG and childhood obesity was no longer significant.

Maternal hyperglycaemia is also likely to be an independent predictor of the risk of childhood obesity. Studies in Pima Indian populations of mothers with frank type 2 diabetes during pregnancy [26, 27] suggest that their children have a greater chance of childhood obesity than those who are pre-diabetic and that these effects are independent of maternal obesity. Gestational diabetes (GDM) [28] is also associated with childhood adiposity. In the context of GDM, maternal glucose levels, which are responsive to therapy, show a positive direct correlation with infant adiposity [29], suggesting that this could potentially be a direct impact of intrauterine exposure.

Data from various disparate populations also suggests that the combination of these two independent risk factors, maternal hyperglycaemia and maternal obesity, has additive effects on the risk of childhood adiposity and childhood BMI [29–31].

A further commonly observed antenatal exposure that correlates strongly with the risk of childhood obesity is maternal smoking [32–34]. Data from a meta-analysis suggests a linear positive association between the number of cigarette smoked and childhood obesity; however, this association may be subject to confounding by the effects of socioeconomic factors and maternal obesity [35].

Maternal stress during pregnancy is also frequently suggested as a factor contributing to the risk of obesity during childhood [36]. A longitudinal prospective study reported that exposure to higher placental cortisol releasing hormone levels in utero was associated with reduced birth weight, followed by a rapid increase in BMI in infancy [36]. However, the observed trend was not directly associated with higher maternal cortisol levels. Further advances in understanding the impact of maternal stress during pregnancy would be helped by the development of more stable direct markers of maternal stress.

### Limitations of Cohort Studies

Interpretation of cohort studies suggesting links between the intrauterine environment and childhood obesity are made more complex as most are from high-income settings, such as Europe and the USA, whereas other settings that have high rates of childhood and adolescent obesity, such as India, Micronesia, Polynesia and Brazil [37] are relatively under-represented. Loss to follow-up is also problematic in many cohort studies and is intrinsically linked to the possibility of introducing bias. The long lead times between pregnancy and the emergence of childhood obesity phenotypes make some loss inevitable; however, many large-scale studies achieve follow-up for fewer than half of their original participants [38]. Other problems of interpretation include heterogeneity in outcome measures between studies, for example the age of measurement of childhood obesity and the parameters used, and self-reported measures such as BMI or birth weights.

Given the inherent difficulties with deriving clear mechanistic insights from human cohort or population-based studies, much of the evidence regarding the molecular pathways linking the intrauterine environment with later childhood obesity is derived from animal models. Animal models can be designed to control for the complex interactions between genetic factors and the postnatal environment and hence are an invaluable source of insight into the specific contribution of antenatal environmental factors in programming childhood obesity risk.

### Evidence from Animal Models

The association between suboptimal intrauterine environments and offspring obesity has been explored extensively, primarily in rodent-based models. The use of animal models has the clear advantage of removing the influence of genetic background and allowing for precise control over the interventions studied. Additionally, the phenotype of the offspring beyond simple anthropometric measures can be extensively unpacked to give mechanistic insights. A number of maternal interventions have been studied to model suboptimal intrauterine environments. Much research effort has focused on maternal diet, as a commonly observed adverse factor in human populations that is easy to model in animals. Recent work has focused on sex differences in offspring outcomes, with some studies suggesting that maternal obesity impacts the risk of obesity more in male than female offspring [39, 40]. Other environmental stressors have also been shown to increase the risk of offspring obesity in animal models, included maternal fragmented sleep [41] and intrauterine hypoxia [42]. By contrast, interventions that improve offspring obesity outcomes in animal models have also been tested, including maternal exercise [43] and dietary fish oil supplementation [44].

The use of animal models allows the antecedents of later offspring obesity, such as foetal overgrowth and placenta nutrient transport to be closely interrogated. A pathway of maternal obesity leading to accelerated placental nutrient transport via upregulation of glucose transporter-1 (GLUT1), sodium-coupled neutral amino acid transporter 2 (SNAT2) and TPM System A and L amino acid transporter activity, resulting in foetal overgrowth, has been defined using mouse models of high-fat/high-sugar diet [45, 46]. In this model, correction of maternal circulating adiponectin levels in late gestation appears to reduce the risk of adverse metabolic phenotypes in offspring, leading to the possibility that maternal adiponectin represents a causal link between maternal and offspring obesity [40]. Multiple other contributing mechanistic insights from animal models into the intrauterine programming of offspring obesity have been identified, as detailed below, but causal links are particularly difficult to establish.

### Epigenetic mechanisms

It is well-established that the intrauterine environment can influence the epigenome of the offspring. A key example is modification of methylation at the Zfp423 promoter in response to high-fat maternal diet. The Zfp423 promoter possesses rich CpG sites and islands, making it a key developmental gene, particularly in developing adipose tissue. Reduced DNA methylation of Zfp423, via the histone modification H3K27me3, is associated with higher Zfp423 expression in adipose tissue and enhanced adipogenesis. In the offspring of dams fed a high-fat diet during pregnancy, there is decreased gene methylation not only of zinc-finger proteins, such as Zfp423 but also key adipogenic transcription regulators such as CCAAT enhancer binding protein B [47, 48]. Another study, also utilising a mouse model of gestational high-fat maternal diet, showed that increased leptin mRNA expression in the offspring was caused by increased acetylation and decreased methylation of histone H3K9 in the adiponectin promoter, alongside increased methylation of histone H4K20 in the leptin gene. These epigenetic effects are associated with insulin resistance and hyperlipidaemia in the offspring [49], providing a plausible mechanism linking maternal and offspring obesity.

### Adipose tissue development

Adipose tissue plays a key role in maintaining metabolic homeostasis. Evidence suggests that offspring adipogenesis can be disrupted by suboptimal intrauterine environments, therefore programming metabolic dysfunction and downstream effects including obesity. Maternal obesity increases foetal adipogenesis in white adipose tissue, while impairing the development of brown adipose tissue, thus dysregulating fat storage and disposing towards obesity [48, 50]. Further evidence suggests that maternal obesity upregulates white adipogenic markers such as resistin in foetal brown adipose tissue, while regulators of brown adipogenesis such as uncoupling protein-1 (UCP1) and PR domain containing 16 (PRDM16) are downregulated [51]. Visceral epididymal adipose tissue is also expanded in offspring from mothers exposed to high-fat diets. Adipose expansion is associated with increased expression and activity of stearoyl-CoA desaturase-1 (Scd1), which is a key component in fatty acid metabolism. This increase in Scd1 was linked to reduced DNA methylation in the Scd1 promoter, reinforcing the idea that the maternal diet programmes program adipose tissue development via the uterine environment [52].

Conversely, evidence from models of maternal dietary restriction also suggests that offspring adipocyte differentiation and maturation can be impacted. In particular, evidence from a low-protein maternal diet rat model shows that miR-483-3p upregulation is associated with inhibited adipocyte differentiation and maturation, leading to altered insulin sensitivity and triglyceride deposition, which are precursors of obesity in offspring [53].

Foetal brain development, in particular development of the hypothalamus, is another possible opportunity for programming of childhood obesity during intrauterine life. Using a mouse model of mothers fed a high-fat diet during pregnancy resulting in hyperphagic offspring with obesity, altered neuronal development in the arcuate nucleus (ARC) of the hypothalamus has been demonstrated [54]. Dysregulation of the development of neuropeptide Y (NPY) neurons in these animals leads to altered control of energy homeostasis and appetite [54]. Hypothalamic insulin resistance in the developing foetal brain has also been demonstrated in a further mouse model of high-fat maternal diet, resulting in abnormal offspring energy homeostasis and feeding regulation into adulthood [55]. This provides a powerful potential mechanism for developmental programming of appetite and hence intrauterine programming of offspring obesity.

Work in animal models has also given rise to other putative mechanisms of developmental programming of offspring obesity. These include reduced mtDNA copy number during embryonic development [56] and intrauterine exposure environmental obesogens, such as bisphenol A (BPA) [57].

## Preventing Childhood Obesity

While the mechanisms underlying intrauterine programming of childhood obesity remain only partially understood, there is nonetheless an urgent need to develop safe and effective interventions to prevent childhood obesity. Much research effort has gone into disentangling the mechanistic aspects, including separating out the relative contributions of genetics, postnatal environment and the antenatal environment. However, it seems highly likely that a synergistic combination of all three aspects has led to the current global increases in childhood obesity, and the development of interventions should not be delayed while these aspects are further researched.

### Identifying Children at Risk

An important first step towards intervening to prevent intrauterine programming of childhood obesity is identifying the pregnancies at highest risk. It is well known that women diagnosed with gestational diabetes are more likely to have children affected by obesity in early childhood (2–5 years) [58]. Maternal obesity has also been directly linked to increased risk of overweight/obesity and fatty liver in childhood [59]; however, these factors alone do not have sufficient predictive power to be clinically useful in identifying children at risk, even gestational and postnatal factors are combined in optimal models [60]. In the absence of predictive models that could usefully be applied during pregnancy to identify women at highest risk of having infants with obesity in childhood, the majority of intervention studies have focused either on whole population level interventions or on very broad risk groups, such as mothers with high BMI.

### The Impact of Exercise Interventions in Pregnancy

Several studies have explored whether exercise-based interventions during pregnancy might reduce the risk of childhood obesity. Murine models suggest that exercise in mothers with obesity prior to and during gestation improves maternal insulin resistance, reduces placental lipid deposition and improves insulin sensitivity in offspring [43, 61–63]. However, there are relatively few exercise-only intervention studies conducted during pregnancy in humans and followed up into childhood (reviewed in [64]). Those that do exist suggest that exercise during pregnancy may reduce the risk of childhood obesity in the offspring [65, 66]. However, there is insufficient data from interventional studies to assess the type, intensity and duration of exercise required to have meaningful impact of the risk of childhood obesity.

### The Impact of Dietary Interventions in Pregnancy

Dietary modification and nutritional supplementation during pregnancy have shown promising results in modulating maternal metabolic parameters for example GWG and insulin resistance. Observational studies of maternal adherence to a ‘Mediterranean diet’ suggest that increased dietary scores are associated with reduced BMI in mid-childhood (4–7 years) [67]. However, follow-up studies from clinical trials suggest that, like exercise interventions, dietary modification translates less well to sustained improvements in offspring adiposity. The ROLO study in Ireland showed that randomisation to a low GI diet during pregnancy reduces neonatal thigh circumference, but not other anthropometric measurements or longer-term adiposity risk [68], with similar results obtained from other populations [69].

Specific dietary modifications and supplements during pregnancy have also been studied in the hope of reducing the risk of subsequent childhood obesity. Among the most widely studied interventions is supplementation with n-3 long-chain polyunsaturated fatty acids, which pre-clinical and observational studies suggest might be associated with a lower risk of childhood obesity [70]. However, a systematic review of clinical trial of n-3 LCPFA supplementation in pregnancy or lactation suggests no significant benefit [71]. Based on similar rationale, a randomized-controlled trial in a low-income African-American cohort in the US showed that docosahexaenoic acid (DHA) supplementation in pregnant women led to lower levels of perceived stress and stress hormones in the third trimester [72], but follow-up data are not available to assess the direct impact on childhood obesity. Observational data from a large US cohort suggest that prenatal vitamin supplementation is also an ineffective intervention to reduce childhood obesity risk [73].

### Combined Lifestyle Interventions

Combined interventions during pregnancy to limit childhood obesity have been trialled in a variety of populations. The pragmatic approach of tackling diet, exercise and other aspects of lifestyle as a combined package of interventions is intuitively attractive in terms of maximising the benefit of each aspect. The UPBEAT (UK pregnancies better eating and activity trial) study trialled a DVD-based exercise schedule and diet changes in women with BMI > 30 kg/m^2^ [74], an intervention that successfully reduced adiposity in the 6-month old infants. However, by 3–5 years of age, ~ 30% of children were retained in the study, and there was no longer a significant impact of the antenatal intervention in reducing childhood adiposity [75]. The LIMIT trial similarly applied an antenatal diet and lifestyle package, achieving a follow-up rate of ~ 66% of eligible children by 3–5 years of age; however, neither adiposity nor childhood growth was impacted by the intervention [76]. A recent individual-patient level meta-analysis including data from 6 original trials of combined diet/lifestyle antenatal interventions in women who were overweight or obese showed no effect on childhood BMI [38]. Vitamin D plays a significant role in insulin resistance and glucose homeostasis, and decreased vitamin D availability is associated with higher BMI [77, 78]. The impact of vitamin D supplementation in pregnancy was tested in the DALI (combining vitamin D supplementation and lifestyle interventions) study, which recruited women with BMI > 29 kg/m^2^. At birth, the combined DALI intervention was associated with reduced neonatal adiposity, but reduced sedentary time was the main mediator of reduced insulin in the neonates [79]. Despite these promising results, no childhood follow-up data are available to directly assess the impact of the DALI interventions on the risk of obesity.

### Other Possible Interventions

The underwhelming impact of antenatal combined lifestyle inventions has led researchers to explore other novel strategies that might be more effective in combating childhood obesity. Such interventions include mindfulness and emotional behaviour training to reduce maternal stress and over-eating during pregnancy [80] and the implementation of mobile apps to promote healthy lifestyle changes during pregnancy [81, 82]. Tracking devices to help pregnant women understand and self-modify their lifestyle factors have also been trialled in recent years [83]. While the primary trial outcomes for these newer interventions, including reduced GWG and increased physical activity during pregnancy, have generally been promising, data are currently lacking on whether they have any impact of obesity rates in childhood.

### Postnatal Interventions

While intervening antenatally may be ideal to prevent metabolic alterations that can lead to childhood obesity, studies nonetheless suggest that significant benefit may be gained from postnatal interventions in children at risk. School-aged French Polynesian children (a population with one of highest rates of childhood obesity in the world) had slower weight gain compared to a non-intervention group of children from a neighbouring island in response to simple diet/lifestyle-based measures [84]. The efficacy of lifestyle interventions to prevent childhood obesity at a family level has also been demonstrated [85], highlighting the importance of involvement of family members in any strategy involving long-term alterations to lifestyle. Exercise is likely to be beneficial both in pregnancy and in childhood to prevent obesity, with cardiovascular exercise and resistance training both having independent and cumulative beneficial effects [86]. While whole population level strategies, for example restriction on advertising high fat, sugar and salt (HFSS) products, may also be effective in lowering childhood obesity rates [87], there is clear scope for the development of targeted interventions for children at risk. For post-natal interventions to be maximally effective, identification of children at highest risk based on antenatal factors should be prioritised.

## Conclusions

**Fig. 1:**
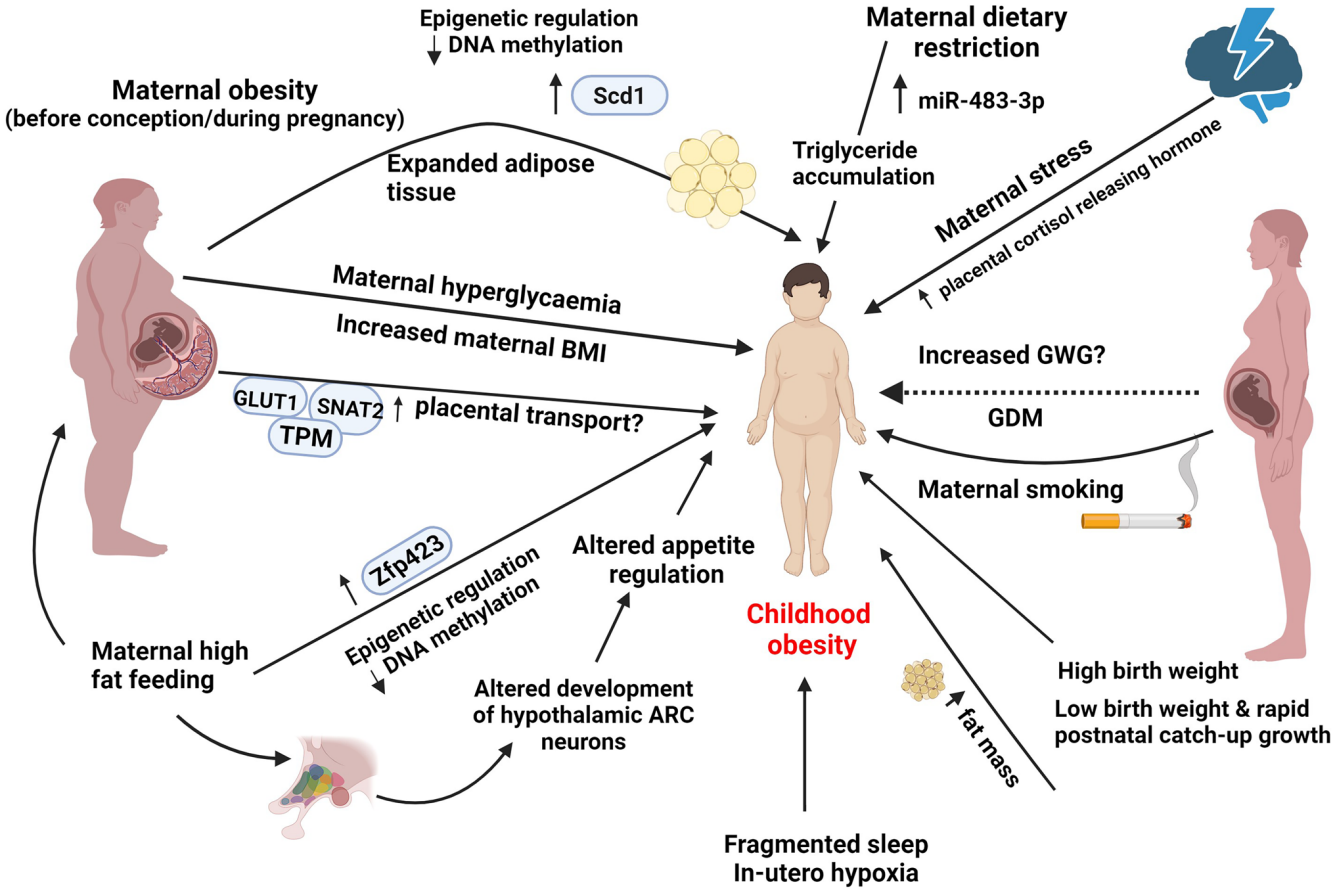
Summary of in utero and early post-natal factors increasing offspring obesity risk (human and animal model evidence). The figure shows a summary of factors and potential mechanisms associated with offspring obesity and adiposity. These include maternal obesity (via adipose tissue expansion and associated epigenetic changes), maternal high fat feeding (via dysregulation of hypothalamic appetite neurons and epigenetic modifications), maternal hyperglycaemia, GDM, increased maternal BMI, maternal smoking, maternal stress (via high placental cortisol releasing hormones), low birth weight in combination with postnatal catch up growth and high birth weight. Previously published material used in this figure are as follows: (15, 16, 17, 18, 19, 20, 21, 22, 26, 27, 29, 32, 33, 34, 36, 41, 42, 45, 46, 47, 48, 52, 54)

Suboptimal intrauterine environments contribute significantly to the risk of childhood obesity through several different pathways (summarised in Fig. 1). Maternal metabolic challenges, for example obesity and insulin resistance, contribute to the risk of foetal overgrowth and subsequent adiposity in childhood. The suboptimal intrauterine environment may lead to epigenetic alterations in metabolic tissues, particularly of expression of genes responsible for adipose tissue deposition and expansion in the offspring. The risk of childhood obesity may also be increased due to central dysregulation of energy homeostasis and appetite regulation (via dysregulation of NPY/AgRP and POMC neurons in the ARC), emphasising the importance of the protecting the developing foetal brain from metabolic alterations during pregnancy where possible. Some further evidence suggests that maternal exposure to environmental obesogens can disrupt lipid metabolism in the foetus, which could potentially contribute to altered lipid handling and hence risk of obesity.

Childhood obesity is increasing by ~ 50 million cases every 5 years [37] and is an antecedent of chronic health conditions including type 2 diabetes, hypertension, atherosclerosis, joint problems and hypercholesterolaemia [88–90]. There is increasing evidence underlying the concern that childhood obesity has a transgenerational impact. Children with obesity are likely to have obesity as adults and thus transmit the increased risk of obesity to future generations, via the intrauterine programming mechanisms discussed here and via other mechanisms. To protect the long-term health of populations, further research is urgently needed to find effective means of identifying and treating those at highest risk of childhood obesity as early in the transgenerational cycle as possible.
